# Comparison of 10-day sequential therapy with 7-day standard triple therapy for *Helicobacter pylori* eradication in inactive peptic ulcer disease and the efficiency of sequential therapy in inactive peptic ulcer disease and non-ulcer dyspepsia

**DOI:** 10.1186/s12876-015-0401-4

**Published:** 2015-12-03

**Authors:** Chung-Chuan Chan, Nai-Hsuan Chien, Chia-Long Lee, Yi-Chen Yang, Chih-Sheng Hung, Tien-Chien Tu, Chi-Hwa Wu

**Affiliations:** Division of Gastroenterology, Department of Internal Medicine, Hsinchu Cathay General Hospital, Hsinchu, Taiwan; Committee of Medical Research and Education, Hsinchu Cathay General Hospital, Hsinchu, Taiwan; Division of Gastroenterology, Department of Internal Medicine, Cathay General Hospital, 280, Section 4, Jen-Ai Road, Taipei, 10650 Taiwan; School of Medicine, Fu Jen Catholic University, New Taipei, Taiwan; Department of Internal Medicine, School of Medicine, College of Medicine, Taipei Medical University, Taipei, Taiwan

**Keywords:** Helicobacter pylori, Inactive Duodenal ulcer, Non-ulcer dyspepsia, Sequential therapy, Triple therapy

## Abstract

**Background:**

Eradication rates of standard triple therapy for *Helicobacter pylori* infections have decreased in recent years due to a worldwide increase in bacterial resistance. Sequential therapy has the advantage of a two-phase treatment regimen and achieves a superior result for *H. pylori* eradication in peptic ulcer disease. However, no study has yet compared the efficacy of sequential therapy for *H. pylori* eradication exclusively in inactive duodenal ulcer (iDU) or non-ulcer dyspepsia (NUD).

**Method:**

We retrospectively recruited 408 patients with endoscopic proven iDU (170 patients) or NUD (238 patients) infected with *H. pylori*. Patients with iDU were assigned into two groups: iDU triple therapy group, 44 patients treated with 40 mg pantoprazole, 1000 mg amoxicillin and 500 mg clarithromycin, twice daily for 7 days; iDU sequential therapy group, 126 patients treated with 40 mg pantoprazole and 1000 mg amoxicillin, twice daily for the first 5 days, followed by 40 mg pantoprazole, 500 mg clarithromycin and 500 mg tinidazole, twice daily for the next 5 days. All patients with NUD were treated with sequential therapy and assigned as the NUD sequential group. Post-treatment *H. pylori* status was confirmed by a ^13^C-urea breath test.

**Result:**

The eradication rates of intention-to-treat (ITT) and per-protocol (PP) analysis were 77.3 % (95 % CI 64.9–89.7 %) and 85.0 % (95 % CI 73.9–96.1 %) in the iDU triple therapy group and 87.3 % (95 % CI 81.5–93.1 %) and 92.4 % (95 % CI 87.6–97.2 %) in the iDU sequential therapy group. The overall eradication efficacy was superior in the sequential group than in the triple group, both with ITT analysis (83.5 % *vs.* 77.3 %, *P* = 0.29) and PP analysis (88.1 % *vs.* 85.0 %, P = 0.57). Eradication rates for ITT and PP analysis were 81.5 % (95 % CI 76.6–86.4 %) and 85.8 % (95 % CI 83.5–88.2 %) in the NUD sequential therapy group. Eradication rate was statistically better in the iDU sequential therapy group than the NUD sequential therapy group according to per protocol analysis (*P* = 0.04). Eradication rate was not significantly different between the iDU sequential- and iDU triple therapy groups according to protocol analysis (*P* = 0.14).

**Conclusion:**

The sequential regimen has a better eradiation rate in the iDU group than in the NUD group. There is no statistically difference between 10-day sequential therapy and 7-day standard triple in iDU group.

## Background

*Helicobacter pylori* (*H. pylori*) has been proved to be a major cause of chronic gastritis, and peptic ulcer disease [1, 2]. Furthermore, *H. pylori* infection is also a crucial cause of gastric cancer [3, 4] and is associated with an increased risk of gastric mucosa-associated lymphoid tissue (MALT) lymphoma [5]. WHO has categorized *H. pylori* as a class I carcinogenic agent in humans; therefore, its eradication has been an important step in the treatment of peptic ulcer disease and prevention of gastric malignancy [6–8].

Treatment of *H. pylori* has been evolving rapidly over the past two decades and several regimens have been proposed to maintain or even increase eradication rates. When first introduced, the now standard triple therapy using proton pump inhibitors, amoxicillin, and clarithromycin, was popular and recommended as first-line therapy for *H. pylori* in Asia and other regions of the world [7–9]. The eradication rates of this regimen, however, have declined below 80 % as observed in many of the latest studies because of increasing drug resistance, mostly to clarithromycin [10–12]. Several approaches have been proposed to overcome the low eradication rates. Sequential therapy was first proposed by Zullo *et al*, in Italy [13]. This two-phase treatment regimen, which involves a proton pump inhibitor plus amoxicillin for the first 5 days followed by a proton pump inhibitor plus clarithromycin and tinidazole or metronidazole for a further 5 days, achieved better results than standard triple therapy [14–16].

Many studies have proved that successful eradication of *H. pylori* substantially reduces the recurrent rate of duodenal ulcers [17, 18] and its recommendation has a worldwide consensus [7–9]. However, no study has yet demonstrated the efficacy of sequential therapy for *H. pylori* eradication exclusively in an inactive duodenal ulcer (iDU). On the other hand, a significant portion of non-ulcer dyspepsia (NUD) patients are infected with *H. pylori* [19] and its eradication improved dyspeptic symptoms. [20] An early study, which compared triple therapy with ranitidine bismuth citrate based quadruple therapy in treatments between peptic ulcer disease (PUD) and NUD patients, revealed better eradication results in PUD [21]. With a similar regimen in another study, there was no convincing evidence to imply that NUD patients responded to *H. pylori* eradication treatments differently from those with PUD [22].

The aim of our study was to compare the efficacy of currently used two-phase sequential therapy with standard triple therapy for *H. pylori* eradication in patients with iDU and the efficiency of sequential therapy in iDU and NUD in the Taiwanese population.

## Methods

### Study population and intervention

We enrolled consecutive patients with endoscopically proven iDU or NUD who were infected with *H. pylori*, which is defined as a positive rapid urease test (CLOtest; Kimbery-Clark, Roswell, GA 30076 USA) from the gastroenterology clinic in one medical center in Taipei, Taiwan. All patients were >18 years of age and had never received treatment for *H. pylori*. Additional exclusion criteria included: (i) consumption of antibiotics, non-steroid anti-inflammatory drugs, proton pump inhibitors (PPI), H2-receptor antagonists, or bismuth salt during the previous four weeks; (ii) allergy or contraindications to antibiotics or PPI; (iii) previous gastric surgery; (iv) severe concomitant cardiopulmonary disease or serious hepatic/renal dysfunction or malignancy; and (v) pregnancy or lactation.

Patients received esophagogastroduodenoscopy (EGD; Olympus, GIF-XP 260) before enrolment to determine iDU or NUD. The study was approved by the Institutional Review Board of the Cathy General Hospital. The trial registration number is CGH-P104077, and the registration date is September 30,2015. Informed consent was obtatined from all patients before EGD.

### Patient selection and *H. pylori* detection

An inactive duodenal ulcer was defined as an endoscopic inspection of a white scar longer than 3 mm with converging folds, located over the duodenal bulb region. Patients with findings of coexisting active ulcers were excluded. Non-ulcer dyspepsia patients were defined as having clinical symptoms of persistent pain or discomfort focused over the epigastric region for at least one month and no abnormality could be detected during endoscopic inspection or during a normal abdominal ultrasound examination. One biopsy specimen was obtained from at least 2 cm away from the pylorus along the greater curvature side of the antrum for a rapid urea test. *H. pylori* infection was diagnosed if the rapid urea test was positive.

### Therapy protocol

Patients with iDU were assigned into 2 groups according to a physician’s discretion: the iDU triple therapy group (hereafter, the iDU triple group) contained 44 patients who received a triple therapy regimen: 40 mg pantoprazole, 1000 mg amoxicillin, and 500 mg clarithromycin, twice daily for 7 days. The iDU sequential therapy group (hereafter, the iDU sequential group) contained 126 patients and they received a sequential therapy regimen: 40 mg pantoprazole and 1000 mg amoxicillin, twice daily for the first 5 days, followed by 40 mg pantoprazole, 500 mg clarithromycin and 500 mg tinidazole, twice daily for the next 5 days. All 238 patients with NUD were treated with a sequential therapy regimen and assigned to the NUD sequential group.

### Post treatment measurement

Results of *H. pylori* status after eradication therapy were determined using a ^13^C-urea breath test (^13^C-UBT). When patients had persistent epigastric symptoms, follow-up endoscopy was performed to make sure there were no newly developed lesions. Assessment of *H. pylori* status again used a rapid urease test (CLO). The ^13^C-UBTs were performed at least 2 months apart and from the date at the end of therapy. The UBT was performed after an overnight fast. A baseline breath sample was obtained and then 75 mg of ^13^C urea with 1.5 g of citric acid was administered as an aqueous solution. The second breath sample was collected 30 min after the intake of test solution. The result was defined positive if the difference between the baseline sample and the 30-min sample exceeded 4.5 per mil of ^13^CO_2_. The sensitivity and specificity values of the UBT were reported as 94.7 % and 95.7 %, respectively [23]. Therapy compliance and drug adverse effects were assessed by personal interview after the end of treatment.

### Statistical analysis

We used intention-to-treat (ITT) and per-protocol (PP) analysis in assessment of the eradication efficacy. Enrolled eligible patients who started medication were all included in the ITT analysis regardless of the correct protocol or compliance. Patients who did not take at least 80 % of the medication or who had incomplete treatment were excluded from PP analysis.

We compared continuous variables with the Student’s test and presented an arithmetic mean and standard deviations. Qualitative variables were analyzed with the chi-square test and presented as percentage and 95 % confidence intervals (95 % CI). All statistical tests were two-sided and all P values <0.05 were considered statistically significant. The analyses were performed using SPSS for Microsoft Window (version 18; SPSS Inc., Chicago, IL, USA).

## Results

### Patients

**Fig. 1:**
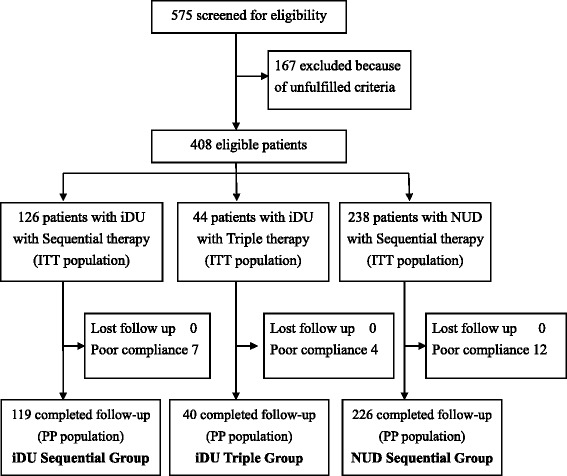
Trial flowchart scheme

From September 2007 to June 2010, we recruited 408 patients with endoscopic proven iDU or NUD and who were infected with *H. pylori.* Figure 1 shows the flow diagram of patients during the protocol. A total of 170 patients were diagnosed as iDU patients and 238 patients were diagnosed as NUD patients. Of the 170 iDU patients, 126 were assigned to the iDU sequential group and the remaining 44 patients were assigned to the iDU triple group. All 238 NUD patients were assigned to the NUD sequential group. Patients of these 3 groups all went through the complete protocol and 7 patients (5.6 %), 4 patients (9.1 %), and 12 patients (5.0 %) of the iDU sequential group, iDU triple group, and NUD sequential group, respectively, did not take the complete regimen of medication. Comparison of clinical characteristics and eradication rates between the iDU sequential group *vs.* the iDU triple group and the iDU sequential group *vs.* the NUD sequential group were performed with both ITT and PP analysis.

### Eradication rates of *H. pylori*

Demographic characteristics of the sequential and triple groups are summarized in Table 1. A total of 364 patients (126 of iDU patients and 238 of NUD patients) received the sequential regimen; mean age was 51.0 ± 11.2 y and females were dominant. Forty-four patients received conventional triple therapy; mean age was 52.6 ± 9.5 y and females were dominant. The eradication efficacy was better in the sequential group than in the triple group, both with ITT analysis (83.5 % *vs.* 77.3 %) and PP analysis (88.1 % *vs.* 85.0 %). Neither analysis, however, demonstrated a significant difference in the 2 therapy regimens.

**Table 1:** Baseline demographic and clinical characteristics of the patients

	ITT		PP	
	Triple therapy	Sequential therapy	Triple therapy	Sequential therapy
Number of patients	44	364	40	345
Age (Mean ± SD)	52.6 ± 9.5	51.0 ± 11.2	53.7 ± 9.0	51.1 ± 11.2
Gender (%)				
Male	9 (20.5 %)	135 (37.1 %)	7 (17.5 %)	127 (36.8 %)
Female	35 (79.5 %)	229 (62.9 %)	33 (82.5 %)	218 (63.2 %)
Endoscopic finding				
iDU	44 (100 %)	126 (34.6 %)		
NUD		238 (65.4 %)		
Eradication success	34	304	34	304
Eradication rate	77.3 %	83.5 %	85.0 %	88.1 %
95 % CI	64.9–89.7 %	79.9–87.3 %	73.9–96.1 %	84.7–91.5 %
P-value	0.29		0.57	

*ITT* intention to treat, *PP* per protocol

Demographic characteristics and eradication rate of the iDU sequential group *vs.* the iDU triple group are summarized in Table 2. Females were more dominant in the iDU triple group than in the iDU sequential group. Age was similar between these two groups. The eradication rate of ITT and PP analyses were 77.3 % (95 % CI 64.9–89.7 %) and 85.0 % (95 % CI 73.9–96.1 %) in the iDU triple group and 87.3 % (95 % CI 81.5–93.1 %) and 92.4 % (95 % CI 87.6–97.2 %) in the iDU sequential group. The eradication rate of *H. pylori* in the iDU sequential group was better than in the iDU triple group both with ITT and PP analyses. However, the differences were not significant and P values were 0.22 and 0.14, respectively.

**Table 2:** Characteristics of subjects in ITT and PP and a comparison of the eradication rate between iDU triple and iDU sequential groups

	ITT		PP	
	iDU triple	iDU sequential	iDU triple	iDU sequential
Number of patients	44	126	40	119
Gender (%)				
Male	9 (20.5 %)	57 (45.2 %)	7 (17.5 %)	52 (43.7 %)
Female	35 (79.5 %)	69 (54.8 %)	33 (82.5 %)	67 (56.3 %)
Age (Mean ± SD)	52.6 ± 9.5	52.4 ± 12.3	53.7 ± 9.0	52.4 ± 12.3
Eradication success	34	110	34	110
Eradication rate	77.3 %	87.3 %	85.0 %	92.4 %
95 % CI	64.9–89.7 %	81.5–93.1 %	73.9–96.1 %	87.6–97.2 %
P-value	0.22		0.14	

*ITT* intention to treat, *PP* per protocol, *iDU* inactive duodenal ulcer

Demographic characteristics and eradication rate of the iDU sequential group *vs.* the NUD sequential group are summarized in Table 3. Females were more dominant in the NUD sequential group than in the iDU sequential group. Age was similar between these two groups. The eradication rate of ITT and PP analyses were 87.3 % (95 % CI 81.5–93.1 %) and 92.4 % (95 % CI 88.4–96.4 %) in the iDU sequential group and 81.5 % (95 % CI 76.6–86.4 %) and 85.8 % (95 % CI 83.5–88.2 %) in the NUD sequential group. The eradication rate of *H. pylori* in the iDU sequential group was better than in the NUD sequential group both for ITT and PP analyses. Difference was significant in PP analysis but not so marked in ITT analysis; P values were 0.04 and 0.11 respectively.

**Table 3:** Characteristics of subjects in ITT and PP and a comparison of the eradication rate between iDU sequential and NUD sequential groups

	ITT		PP	
	iDU Sequential	NUD Sequential	iD U Sequential	NUD Sequential
Number of patients	126	238	119	226
Gender (%)				
Male	57 (45.2 %)	78 (32.8 %)	52 (43.7 %)	75 (33.2 %)
Female	69 (54.8 %)	160 (67.2 %)	67 (56.3 %)	151 (66.8 %)
Age (Mean ± SD)	52.4 ± 12.3	50.3 ± 10.7	52.4 ± 12.3	50.4 ± 10.6
Eradication success	110	194	110	194
Eradication rate	87.3 %	81.5 %	92.4 %	85.80 %
95 % CI	81.5–93.1 %	76.6–86.4 %	88.4–96.4 %	83.5–88.2 %
P-value	0.11		0.04	

*ITT* intention to treat, *PP* per protocol, *iDU* inactive duodenal ulcer, *NUD* non-ulcer dyspepsia

### Adverse events and compliance

Table 4 shows the adverse events and their incidences in conventional triple and sequential therapy groups. The overall adverse event rate was higher in the sequential group than in the triple group (36.3 % *vs.* 22.7 %, *P* = 0.08). The highest incidence of adverse events was mouth bitterness in both groups. Patients reported compliances were similar in both the triple and sequential groups (90.9 % *vs.* 94.8 %, *P* = 0.29).

**Table 4:** Adverse events in sequential therapy

	Triple	iDU Sequential	NUD Sequential	Total Sequential
Adverse event	(*N* = 44)	(*N* = 126)	(*N* = 238)	(*N* = 364)
Taste disturbance	6.8 %	7.10 %	7.60 %	7.40 %
Diarrhea	0.0 %	2.40 %	0.40 %	1.10 %
Abdominal discomfort	0.0 %	3.20 %	0.40 %	1.40 %
Skin rash	0.0 %	1.60 %	0.80 %	1.10 %
Nausea	2.3 %	0.00 %	0.80 %	0.50 %
Poor appetite	0.0 %	0.00 %	0.40 %	0.30 %
Dizziness	0.0 %	2.40 %	0.40 %	1.10 %
Mouth bitterness	11.4 %	28.60 %	11.30 %	17.30 %
Loose stool	2.3 %	3.20 %	7.10 %	5.80 %
Cramp	0.0 %	0.80 %	0.00 %	0.30 %
Total	22.7 %	49.20 %	29.40 %	36.30 %
P value (triple *vs* total sequential)		0.08		

*iDU* inactive duodenal ulcer, *NUD* non-ulcer dyspepsia

## Discussion

Standard triple therapy, when first proposed, was demonstrated to have a high eradication efficacy with a success rate over 85 % [24]. Therefore, most therapeutic guidelines from major academic committees worldwide recommended a proton pump inhibitor based triple therapy plus clarithromycin and amoxicillin or metronidazole as the first-line regimen for eradication of *H. pylori* [7–9]. Unfortunately, the eradication rate of this gold standard therapy declined rapidly during the following 10 years toward unacceptable levels [25]. The frustrating outcome of the poor eradication rate led to several new strategies aimed to raise eradication efficacy. Innovative therapeutic approaches included extending therapy duration, the altering of conventionally used antibiotics to novel ones, and the addition of multi-drug regimens.

A two-phase sequential regimen, by adding a fourth drug, has been shown to have promising eradication results in many studies worldwide. However, most of these trials were conducted in patients diagnosed with *H. pylori* infection and without separately analyzed eradication rates in either ulcer related or non-ulcer related groups. Our current work was a trial to compare the eradication efficacy of sequential therapy and conventional triple therapy exclusively in inactive duodenal ulcer and non-ulcer dyspepsia patients. The results demonstrated that a 10-day sequential regimen was superior to conventional triple therapy for eradication of *H. pylori* in treatment naïve patients. Overall, the eradication rate of sequential therapy was 88.1 % with PP analysis and 83.5 % with ITT analysis, which were higher than those of triple therapy (85.0 % with PP analysis and 77.3 % with ITT analysis). The result is similar to that shown in other studies in Western and Asian countries [26–31]. However, in our trial, the eradication rate of sequential therapy did not reach the good category according to the Graham’s report card for grading *H. pylori* therapy [32] (90–95 % intention-to-treat) as with most other studies [15, 33, 34] and did not demonstrate a significant difference compared to triple therapy. One randomized controlled trial in southern Taiwan reported a high eradication rate (92.9 % with PP and 93.2 % with ITT analysis) of sequential therapy [35]. Patients they enrolled had either gastric ulcers or duodenal ulcers (94.3 % of total patients). We suggest the reason why our results did not yield a higher eradication efficacy may be due to the type of gastroduodenal diseases in our patients. Our patient pool only consisted of either inactive duodenal ulcer or non-ulcer dyspepsia patients.

Previous studies found that the eradication rate of standard triple therapy in non-ulcer dyspepsia tends to be lower than that in peptic-ulcer patients [21, 36]. Contrary results are observed in sequential therapy and the success rates are not significantly affected by pathological findings (peptic-ulcer *vs.* non-ulcer dyspepsia) [37, 38]. Our study’s result was different, in that the eradication rate of sequential therapy (Table 3) was better in duodenal ulcer scar patients than in non-ulcer dyspepsia with ITT analysis (87.3 % *vs.* 81.5 %) and with PP analysis (92.4 % *vs.* 85.8 %), which reached a significant difference. Two large studies (DU-MACH [39] and GU-MACH [40]) have therefore looked at the impact of inflammation on H. pylori eradication. Polymorph infiltration in the antrum of patients with inflammation of grades 2/3 was associated with a significantly higher eradication rate when compared with inflammation of grades 0/1. Previous ulcer diseases may induce inflammation processes that cause degradation of the mucus and epithelial layers and altered epithelial permeability. That may allow better penetration of antibiotics from the gastric lumen and better systemic delivery of drugs. Besides, the subtype of H. Pylori that cause ulcer may be more aggressive which has a better response to the antibiotics. Therefore, we speculate that the low eradication rate of sequential therapy in non-ulcer dyspepsia patients tarnished the overall eradication rate of sequential therapy in our 364 patients with either duodenal ulcer scar or non-ulcer dyspepsia. Although eradication of *Helicobacter Pylori* is both suggested in patient with duodenal ulcer scar or non-ulcer dyspepsia, the difference in eradication rate could provide us a better outcome predication to discuss with the patient.

Although the difference was not statistically significant, subgroup analysis revealed a superior eradication rate of sequential therapy in duodenal ulcer scar patients (Table 2; ITT, 87.3 % *vs.* 77.3 %; PP, 92.4 % *vs.* 85.0 %). Sequential therapy also achieved the acceptable effectiveness category (86–89 %, ITT) and was better than triple therapy (unacceptable category: <80 %, ITT) according to Graham’s category [32]. Since many studies have proved that successful eradication of *H. pylori* substantially reduces the recurrent rate of duodenal ulcers, it is recommended by a worldwide consensus. We believe that a 10-day sequential regimen could be a valid alternative therapy in initial treatment for the eradication of *H. pylori* in duodenal ulcer scar patients.

Our data showed that the overall adverse event rate was higher in the sequential group than that in the triple group (Table 4; 36.3 % *vs.* 22.7 %). The highest incidence of adverse events was mouth bitterness in both groups. The sequential group had an even higher frequency of mouth bitterness. Nonetheless, patient-reported compliance was similar in both the triple and sequential therapy groups (90.9 % *vs.* 94.8 %).

We also performed the multivariate analysis to investigate the independent factors predicting eradication failure in this study. Table 5 showed the eradication efficiency has no relationship with age and gender. Besides, the successful eradication group has a significantly higher adverse reaction rate then the failure group. It seemed that the eradication rate was associated independently with the different protocol and presence of ulcer scar.

**Table 5:** Multivariate analysis to investigate the independent factors

	Total (*N* = 408)	Eradication failure (*N* = 70)	Eradication success (*N* = 338)	*p*-value
Age (Mean ± SD)	51.2 ± 11.1	50.7 ± 10.5	51.3 ± 11.1	0.67
Gender (%)				
Male	144	22 (31.4 %)	122 (36.1 %)	0.46
Female	264	48 (68.6 %)	216 (63.9 %)	
Adverse event (%)				
Yes	140	16 (22.9 %)	124 (36.7 %)	0.03
No	268	54 (77.1 %)	214 (63.3 %)	
There is no statistically difference between eradication rate, gender, and age. The success group have more adverse event.				
iDU	Total failure in iDU (N = 26)	Failure in Triple (N = 10)	Failure in Sequential (N = 16)	*p*-value
Age (Mean ± SD)	51.7 ± 9.7	48.5 ± 7.9	53.7 ± 10.4	0.19
Gender (%)				
Male	9	3 (30.0 %)	6 (37.5 %)	0.7
Female	17	7 (70.0 %)	10 (62.5 %)	
Adverse event (%)				
Yes	10	2 (20.0 %)	8 (50.0 %)	0.13
No	16	8 (80.0 %)	8 (50.0 %)	
In iDU group, there is no statistically difference between failure rate, age, gender, and adverse, event.				
Sequential	Total failure in Sequential (N = 60)	Failure in iDU (N = 16)	Failure in NUD (N = 44)	*p*-value
Age (Mean ± SD)	51.0 ± 10.9	53.7 ± 10.4	50.0 ± 11.0	0.26
Gender (%)				
Male	19	6 (37.5 %)	13 (29.5 %)	0.56
Female	41	10 (62.5 %)	31 (70.5 %)	
Adverse event (%)				
Yes	14	8 (50.0 %)	6 (13.6 %)	0.003
No	46	8 (50.0 %)	38 (86.4 %)	
In sequential group, there is no statistically difference between failure rate, age, and gender. However, the adverse rate is statistically higher in iDU group.				

Furthermore, Table 6 shows the cost of each individual regimen. Clarithromycin was the most expensive drug and cost $32.30 in the standard triple therapy regimen, out of a total cost of $51.00. The sequential therapy regimen cost only $47.6. Due to the comparative expense of standard therapy, replacement of it with the less costly sequential regimen would greatly reduce total treatment costs. This economical consideration also favors the use of the latter treatment regimen.

**Table 6:** The therapy dose and cost for triple and sequential therapy

Group	Regimen	Dose		Cost
Triple therapy	Pantoprazole	40 mg	BID × 7	$14.60
	Amoxicillin	1 gm	BID × 7	$4.10
	Clarithromycin	500 mg	BID × 7	$32.30
				Total $51.00
Sequential therapy	Pantoprazole	40 mg	BID × 5	$10.40
	Amoxicillin	1 gm	BID × 5	$3.00
	Pantoprazole	40 mg	BID × 5	$10.40
	Clarithromycin	500 mg	BID × 5	$23.10
	Tinidazole	500 mg	BID × 5	$0.70
				Total $47.60

The present study had a couple of limitations. Firstly, that patients were not randomized to receive either standard triple therapy or 10-day sequential therapy. Patients with iDU were assigned into 2 groups according to a physician’s discretion, which may have introduced selection bias. Therefore, the number in the iDU triple group was too small. However, all patients were prospectively followed up with a standard protocol and were well informed about adverse problems and compliance. Secondly, bacterial culture was not performed in our protocol, and therefore the effect of antibiotic resistance was not able to be assessed. However, the resistance rates of Amoxicillin, Clarithromycin, Metronidazole, and Tetracycline in Cathy General Hospital are 13.9, 27.8, 19.4, and 0 % respectively.

## Conclusion

Despite no statistically significant difference in ITT patients, 10-day sequential therapy for the eradication of *H. pylori* was superior in iDU patients than NUD patients and it reached a significant eradication effectiveness in PP patients. The sequential regimen has a better eradication rate in the iDU group than in the NUD group.

## Abbreviations

*H. pylori*: *Helicobacter pylori*
iDU: inactive duodenal ulcer
NUD: non-ulcer dyspepsia
ITT: intention-to-treat
PP: per-protocol
PUD: peptic ulcer disease
EGD: esophagogastroduodenoscopy
C-UBT: ^13^C-urea breath test
CLOtest: rapid urease test
